# Prognostic impact of left ventricular myocardial work in patients undergoing surgery for primary mitral regurgitation

**DOI:** 10.1007/s10554-025-03386-x

**Published:** 2025-03-29

**Authors:** Takeru Nabeta, Ferande Peters, Hoi W Wu, Aileen Paula Chua, Meindert Palmen, Anton Tomšič, Nina Ajmone Marsan, Jeroen J Bax, Pieter van der Bijl

**Affiliations:** 1 Department of Cardiology, Leiden University Medical Centre, Leiden, The Netherlands; 2 Department of Cardiovascular Medicine, Kitasato University School of Medicine, Sagamihara, Japan; 3 Cardiovascular Pathophysiology Research Unit, University of the Witwatersrand, Johannesburg, South Africa; 4 Department of Cardiothoracic Surgery, Leiden University Medical Centre, Leiden, The Netherlands; 5 Department of Cardiothoracic Surgery, University Hospital Augsburg, Augsburg, Germany; 6 Heart Centre, University of Turku, Turku University Hospital, Turku, Finland; 7 Department of Cardiology, Heart Lung Centre, Leiden, 2300 RC The Netherlands

**Keywords:** Primary mitral regurgitation, Left ventricular function, Myocardial work indices, Mortality

## Abstract

**Purpose:**

Echocardiography-based, left ventricular myocardial work (LVMW) can assess LV function by incorporating LV afterload. This study aims to evaluate the prognostic value of LVMW indices in patients with primary mitral regurgitation (MR) undergoing mitral valve surgery.

**Methods and results:**

A total of 306 patients (mean age 63 ± 12 years, 68% male) with severe, primary MR who underwent surgery, were included. All patients underwent transthoracic echocardiography and LVMW indices were assessed with commercially available ultrasound equipment before surgery. The mean LV global work index (LVGWI) was 1979 ± 537 mmHg% and 130 (42%) patients had impaired LVGWI (≤ 1900 mmHg%). During a median follow-up of 5.0 years (interquartile range, 2.5–8.9), 27 (8.8%) patients died after mitral valve surgery. Patients with impaired LVGWI or LV global longitudinal strain (LVGLS) (≤ 20%) had lower survival rates compared to the group with preserved (*p* < 0.01 and *p* = 0.02, respectively). While the likelihood ratio test suggests that LVGWI ≤ 1900 mmHg% provides additional prognostic information beyond the model including LVGLS (*p* < 0.05) for all-cause mortality, no significant improvement was observed in area under the curve, the C-index, or net-reclassification index.

**Conclusions:**

In patients with severe, primary MR who underwent surgery, impaired pre-operative LVGWI was associated with a higher mortality risk, and may have incremental value beyond LVGLS, but requires further study for validation.

**Supplementary Information:**

The online version contains supplementary material available at 10.1007/s10554-025-03386-x.

## Introduction

Severe, primary mitral regurgitation (MR) is associated with an increased risk of mortality when untreated [1, 2]. Surgical mitral valve (MV) repair is the standard of care for severe, primary MR [3]. Preserved left ventricular (LV) function before surgery predicts a more favorable outcome in patients undergoing MV surgery [4]. Consequently, current guidelines provide a class 1 recommendation for MV surgery in patients with severe, primary MR when the LV ejection fraction (LVEF) decreases below 60% or the LV end-systolic diameter increases to 40 mm or more, regardless of symptoms [3]. 

Although LVEF is widely used as a parameter for the assessment of LV systolic function, it suffers from significant limitations in patients with severe MR. Since LVEF is highly load-dependent, and the LV unloads into the low-pressure left atrium (LA) during ventricular systole, LVEF overestimates LV systolic function in patients with severe MR [5]. LV global longitudinal strain (LVGLS) is a more sensitive maker of systolic function in the context of MR, and is firmly linked to outcomes after surgery for primary MR [6–8]. LVGLS, despite being less load-dependent than LVEF, remains an afterload dependent measure of LV systolic function [9, 10]. 

Non-invasive LV myocardial work (LVMW) incorporates afterload into the quantification of LV systolic function, and may have particular utility in circumstances where the afterload is dynamic, e.g. before and after MV surgery [11]. Previous studies demonstrated that LVMW parameters were independently associated with mortality in patients who underwent cardiac resynchronization therapy [12] and in secondary MR [13]. Echocardiography-derived LVMW may be an even more accurate prognostic maker than LVGLS in patients with primary MR undergoing surgery– a hypothesis which has not been investigated yet. We therefore evaluated the prognostic value of pre-operative LVMW in patients with severe, primary MR undergoing MV surgery.

## Methods

### Patient population

Individuals who underwent MV surgery for severe, primary MR at the Leiden University Medical Center between 2006 and 2021 were included. Patients with hypertrophic cardiomyopathy, previous cardiac surgery or significant aortic valve disease and those who underwent concomitant aortic valve surgery at the time of MV surgery, as well as those who died from the complications directly related to the surgery, were excluded. Patients who underwent MV replacement were also excluded. Patients with suboptimal echocardiographic images deemed unsuitable for the measurement of LVMW (e.g. low image quality or from a vendor that does not support MW calculation) and those without blood pressure measurements at the same day of echocardiography, were also excluded. Demographic and clinical data were collected from the electronic patient files (EPD-vision, Leiden University Medical Center, Leiden, The Netherlands) and retrospectively analyzed. Clinical data included demographic characteristics and comorbidities and were obtained before surgery. Chronic kidney disease was defined as an estimated glomerular filtration rate ≤ 60 ml/min/1.73m^2^. The study complies with the Declaration of Helsinki and was approved by the Institutional Review Board. Due to the retrospective design of this study, the Medical Ethical Committee waived the need for written informed consent.

## Echocardiographic evaluation

All patients underwent transthoracic echocardiography with commercially available ultrasound equipment (Vivid 7, E9 or E95 GE-Vingmed, Horten, Norway) before surgery. Electrocardiography-triggered echocardiographic data were digitally stored in cine-loop format for offline analysis using EchoPAC versions 113, 203 and 204 (GE-Medical Systems, Horten, Norway). LV end-diastolic and end-systolic volumes, LVEF and LA volumes were measured using the Simpson’s biplane method [14] and LV and LA volumes were indexed to body surface area. Right ventricular (RV) function was quantified by tricuspid annular plane systolic excursion (TAPSE). Systolic pulmonary artery pressure was estimated as the sum of the right atrial pressure and the RV end-systolic pressure gradient. Right atrial pressure was estimated based on inferior vena cava diameter and collapse during inspiration [15]. The severity of MR and tricuspid regurgitation (TR) was assessed using a multiparametric approach according to current recommendations [16]. 

LVMW was derived from a vendor-specific package (EchoPAC 204 GE-Medical Systems, Horten, Norway) which integrates LVGLS with sphygmomanometric blood pressure to construct pressure-strain loops during the cardiac cycle [11, 13]. Blood pressure was measured on the same day that echocardiography was performed. LVGLS was measured from the apical four-chamber, two-chamber and long-axis views of the LV and expressed as an absolute value [17]. After LVGLS measurements, the timing of aortic and MV opening and closure as well as blood pressure were entered into the software. Four LVWM parameters were calculated by the software: (1) LV global myocardial work index (LVGWI) was derived from the area within a pressure-strain loop from MV closure to opening, (2) LV global constructive work (LVGCW) was defined as shortening during systole and lengthening during isovolumic relaxation, (3) LV global wasted work (LVGWW) was defined as lengthening during systole and shortening during isovolumic relaxation and (4) LV global work efficiency (LVGWE) was calculated by dividing LVGCW by the sum of LVGCW and LVGWW.

The prognostic thresholds of LVEF (60%) and LVGLS (20%) were derived from previous studies in patients with primary MR [4, 7, 8]. For the LVMW indices, previously-established normal values were used as thresholds: LVGWI 1900 mmHg%, LVGCW 2200 mmHg%, LVGWW 90 mmHg% and LVGWE 96% [9]. 

## Clinical endpoints

The primary endpoint was all-cause mortality after MV surgery. Survival data were collected from municipal civil registries linked to the patients’ medical records and were complete for all patients.

### Statistical analysis

Categorical variables were expressed as numbers and percentages. Continuous data were presented as mean ± standard deviation. Group differences were evaluated using Student’s t-test for continuous variables and the chi-square test or Fisher’s exact test for categorical variables, as appropriate. Cumulative, event-free survival rates were calculated using the Kaplan-Meier method, while the log-rank test was used to compare the risk of events between patient groups, as defined by echocardiographic thresholds. Univariate and multivariate Cox regression analysis was used to identify variables associated with all-cause mortality. Variables with a p-value < 0.05 in the univariate analysis were included in the multivariate models. Moreover, the incremental prognostic value of LVGWI was evaluated by the change in likelihood ratio chi-square value for nested models, when added to baseline models including variables with a p-value < 0.05 in the univariate Cox regression analysis for all-cause mortality. In addition, we compared the area under the curve (AUC) for 5-year mortality, C-statistics, and net reclassification index (NRI) between the model including LVGWI and LVGLS to assess whether the addition of LVGWI showed improved discrimination or reclassification. We also investigated whether LVGWI ≤ 1900 mmHg% was associated with all-cause death when adjusting for atrial fibrillation, systolic pulmonary artery pressure, moderate TR, TAPSE, effective regurgitant orifice area or New York heart association functional class. To assess intra- and inter-class correlation, repeated LVGWI and LVGLS measurements were performed for *n* = 45 patients by a single observer at two different points and by a second observer blinded to the measurements of the first observer. All statistical analyses were performed using SPSS version 29.0 (IBM Corporation, Armonk, NY, USA) and R software version 4.1.1 (R Foundation for Statistical Computing, Vienna, Austria). All p-values were two-sided and values < 0.05 were considered statistically significant.

## Results

### Patient characteristics

**Fig. 1:**
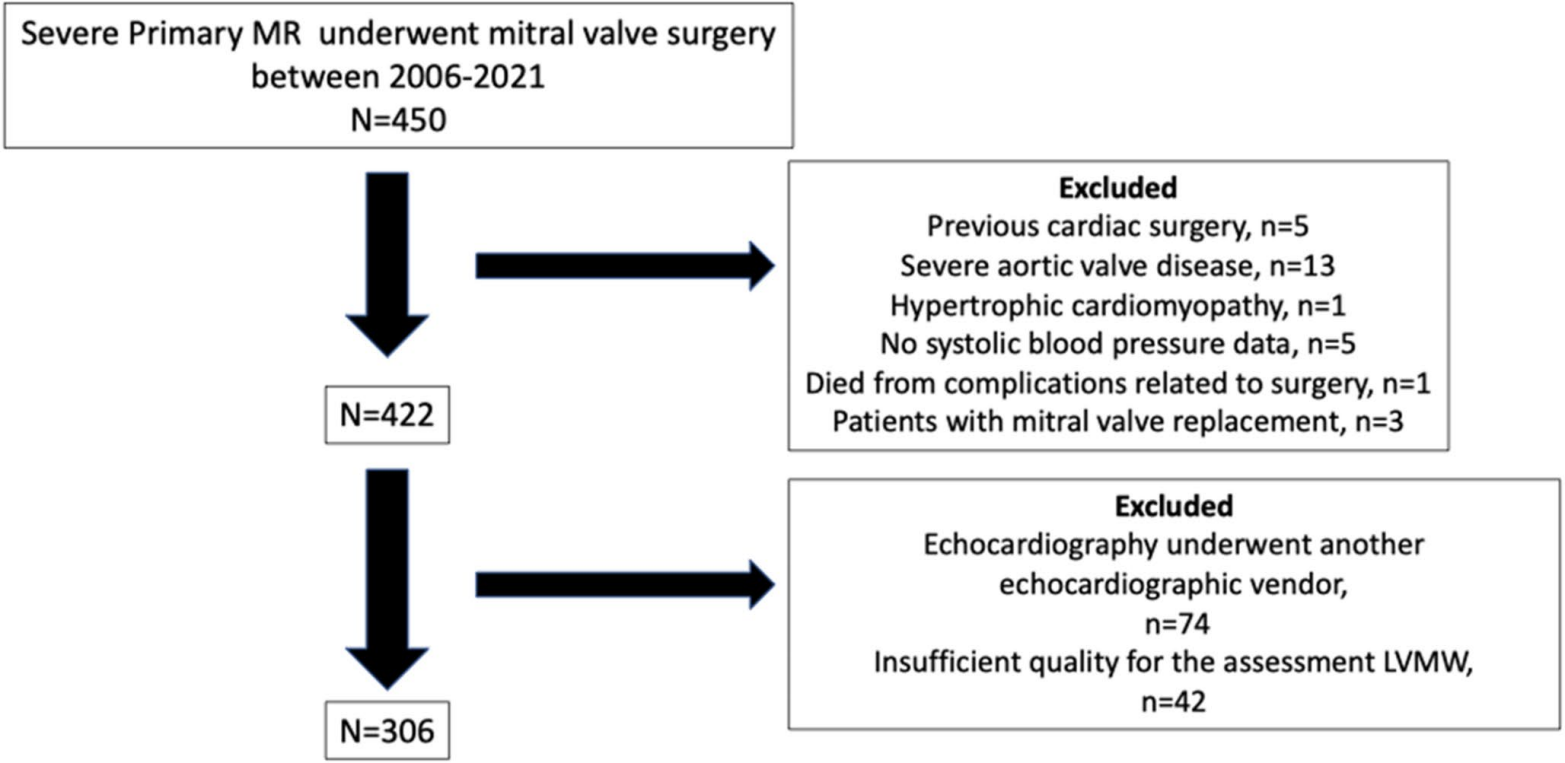
Patient flow MR; mitral regurgitation, LVMW; left ventricular myocardial work

A total of 306 patients (mean age 63 ± 12 years, 68% male) were included (Fig. 1). Pre-operative echocardiography was performed at a median of 49 days (interquartile range, 13–128 days) before surgery. Table 1 shows the baseline characteristics and the differences between patients with LVGWI ≤ 1900 mmHg% and those with LVGWI > 1900 mmHg%. A total of 164 (54%) patients had fibroelastic deficiency and 142 (46%) patients were classified as having advanced myxomatous disease. Chronic kidney disease and atrial fibrillation were more common in patients with LVGWI ≤ 1900 mmHg% than in those with LVGWI > 1900 mmHg%. Concomitant tricuspid valve repair was performed in 148 (48%) patients, and was more commonly performed in patients with LVGWI ≤ 1900 mmHg%. The baseline clinical and echocardiographic characteristics, stratified by the occurrence of death are shown in Supplemental Table 1.

**Table 1:** Baseline characteristics

Characteristic	Overall population *N* = 306	LVGWI ≤ 1900 mmHg% *N* = 130	LVGWI > 1900 mmHg% *N* = 176	*p*-value
Age (years)	63 (12)	66 (11)	61 (11)	< 0.001
Gender (male)	209 (68%)	92 (71%)	117 (66%)	0.425
Etiology of mitral regurgitation				0.017
Fibroelastic deficiency	164 (54%)	80 (62%)	84 (48%)	
Advanced myxomatous disease	142 (46%)	50 (38%)	92 (52%)	
NYHA functional class ≥ II	229 (75%)	109 (84%)	120 (68%)	0.002
Arterial hypertension	121 (40%)	49 (38%)	72 (41%)	0.569
Hypercholesterolemia	19 (12%)	7 (11%)	12 (12%)	0.888
Diabetes mellitus	9 (2.9%)	5 (3.8%)	4 (2.3%)	0.502
Chronic kidney disease	55 (18%)	36 (28%)	19 (11%)	< 0.001
Atrial fibrillation	48 (16%)	40 (31%)	8 (4.5%)	< 0.001
Systolic blood pressure (mmHg)	135 (19)	127 (19)	141 (17)	< 0.001
Diastolic blood pressure (mmHg)	78 (11)	76 (12)	79 (11)	0.023
Heart rate (/min)	75 (16)	78 (19)	72 (13)	0.003
Cardiac surgery				
Tricuspid valve repair	148 (48%)	76 (58%)	72 (41%)	0.002
Coronary artery bypass grafting	41 (13%)	18 (14%)	23 (13%)	0.843

Values are expressed as mean (± SD). LVGWI, left ventricular global myocardial work index; NYHA, New York Heart Association

## Echocardiographic parameters

The pre-surgical echocardiographic parameters are summarized in Table 2. The mean LVEF was 64 ± 8% and the mean LVGLS 19 ± 4%. The mean LVGWI was 1979 ± 539 mmHg% and 130 (42%) patients had a LVGWI ≤ 1900 mmHg%. LVEF and LVGLS were lower in patients with LVGWI ≤ 1900 mmHg% than in those with LVGWI > 1900 mmHg%. Among patients with LVEF > 60% (223, 73%), 75 (34%) patients had LVGWI ≤ 1900 mmHg%. TAPSE was lower and systolic pulmonary artery pressure was higher in patients with LVGWI ≤ 1900 mmHg%, compared to those with LVGWI > 1900 mmHg%. The intra-class correlation coefficient was 0.85 (95%CI 0.74 to 0.91) for LVGWI and 0.95 (95%CI 0.91 to 0.97) for LVGLS. Similarly, the inter-class correlation was 0.89 (95%CI 0.81 to 0.94) for LVGWI and 0.96 (95%CI 0.93 to 0.98) for LVGLS.

**Table 2:** Baseline echocardiographic parameters

Characteristic	Overall population *N* = 306	LVGWI ≤ 1900 mmHg% *N* = 130	LVGWI > 1900 mmHg% *N* = 176	*p*-value	Missing
LVEDV index (ml/m2)	75 (20)	72 (19)	77 (21)	0.037	0 (0%)
LVESV index (ml/m2)	27 (10)	29 (12)	26 (8)	0.014	0 (0%)
LVEF (%)	64 (8)	61 (10)	67 (6)	< 0.001	0 (0%)
LVGLS (%)	19 (4)	16 (4)	21 (3)	< 0.001	0 (0%)
LAVI (ml/m2)	56 (23)	58 (24)	55 (21)	0.237	1 (0.3%)
LVGWI (mmHg%)	1979 (539)	1479 (311)	2349 (333)	< 0.001	0 (0%)
LVGCW (mmHg%)	2360 (585)	1865 (384)	2725 (414)	< 0.001	0 (0%)
LVGWW (mmHg%)	151 (104)	164 (114)	141 (96)	0.062	0 (0%)
LVGWE (%)	92.3 (4.4)	90.1 (5.0)	93.8 (3.0)	< 0.001	0 (0%)
EROA (mm2)	49 (20)	52 (22)	46 (19)	0.020	18 (5.9%)
Regurgitant volume (ml)	62 (24)	63 (23)	61 (25)	0.425	18 (5.9%)
Vena contracta (mm)	7.1 (1.6)	7.2 (1.7)	6.9 (1.5)	0.087	8 (2.6%)
TAPSE (mm)	23 (5)	22 (5)	25 (4.)	< 0.001	2 (0.6%)
Systolic PAP (mmHg)	38 (16)	42 (17)	35 (14)	< 0.001	22 (7.2%)
TR ≥ moderate	71 (23%)	43 (33%)	28 (16%)	< 0.001	0 (0%)

Values are expressed as mean (± SD). EORA, effective regurgitant orifice area; LAVI, left atrial volume index; LVEDV, left ventricular end-diastolic volume; LVEF, left ventricular ejection fraction; LVESV, left ventricular end-systolic volume; LVGLS, left ventricular global longitudinal strain; LVGWI, left ventricular global myocardial work index; LVGCW, left ventricular global constructive work; LVGWW, left ventricular global wasted work; LVGWE, left ventricular global work efficiency; PAP, pulmonary artery pressure; TAPSE, tricuspid annular plane systolic excursion; TR, tricuspid regurgitation

## Survival analysis

**Fig. 2:**
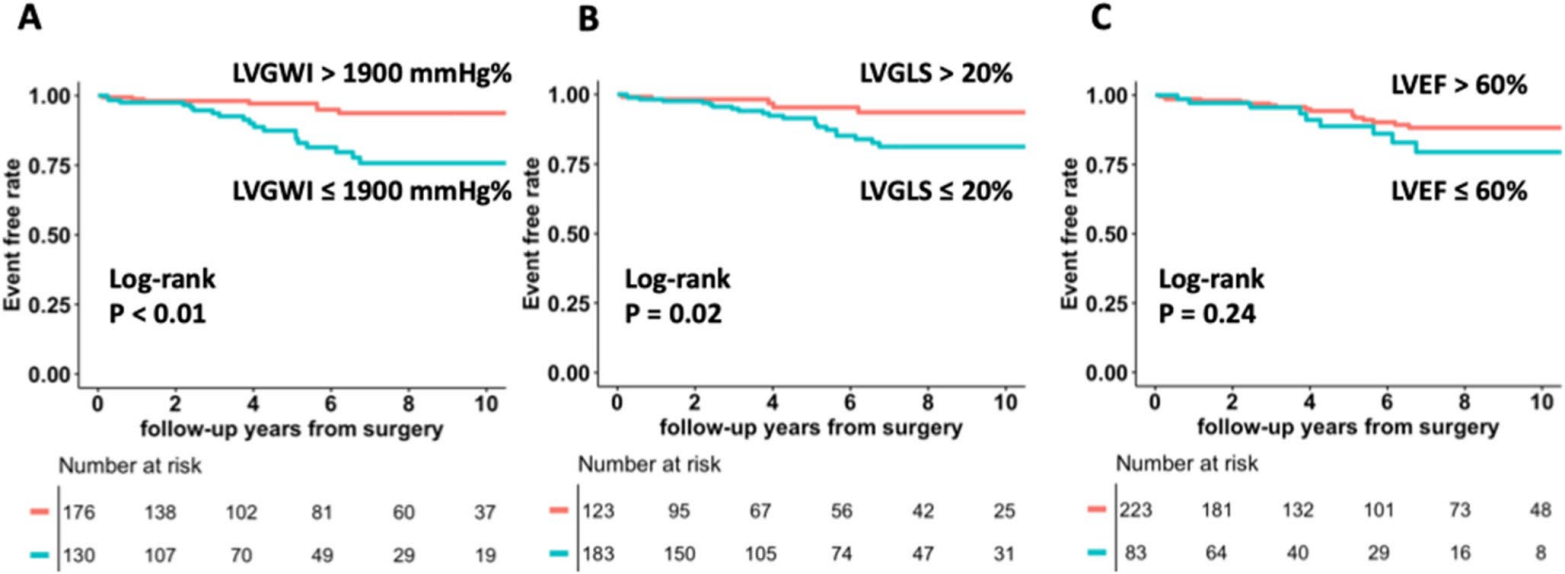
Kaplan-Meier curves for all-cause mortality, stratified according to various left ventricular functional parameters Kaplan-Meier curves for left ventricular global myocardial work index (LVGWI, A), LV global longitudinal strain (LVGLS, B) and LV ejection fraction (LVEF, C) with patients stratified by threshold values previously shown to have prognostic value

**Fig. 3:**
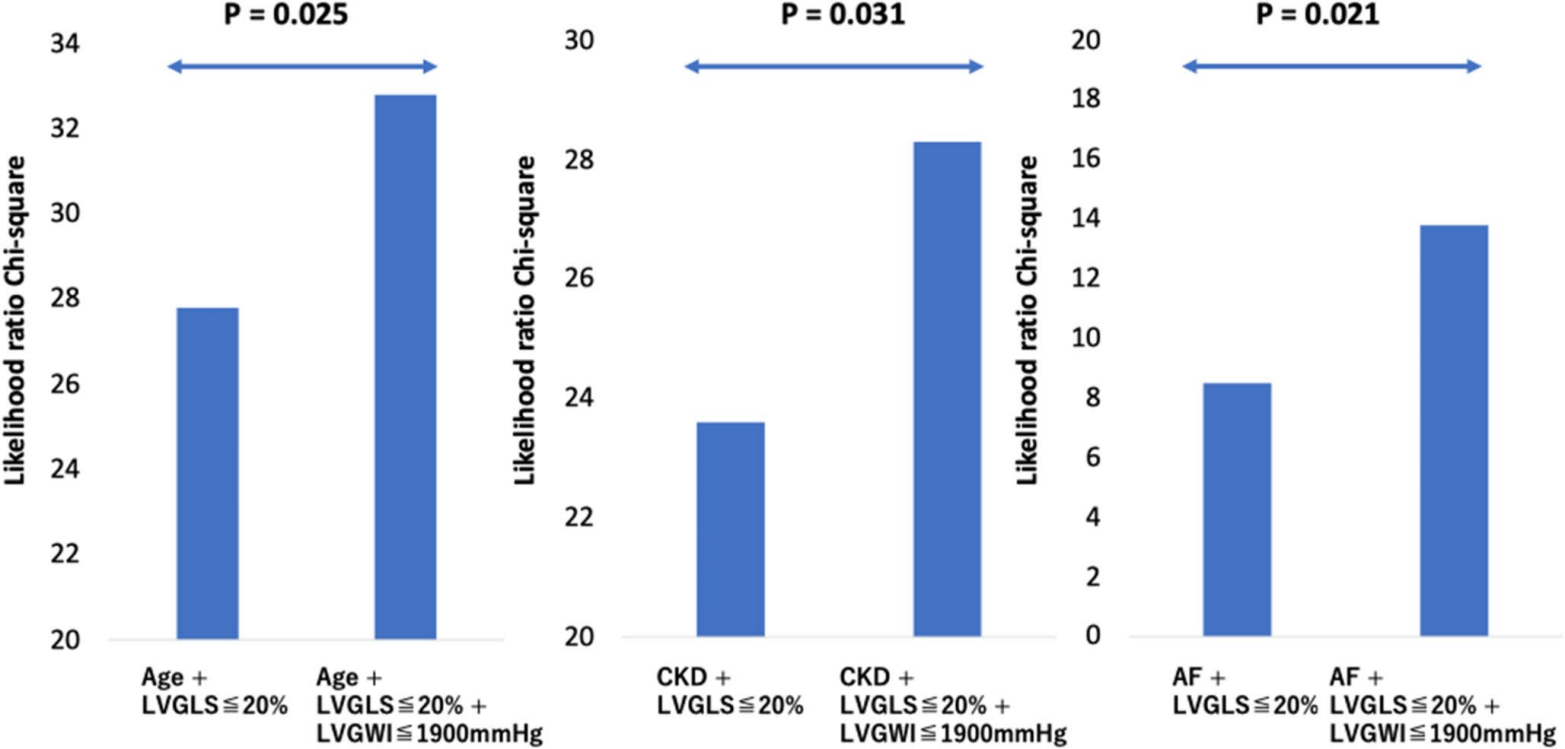
Incremental prognostic value of left ventricular global myocardial work A left ventricular global work index (LVGWI) ≤ 1900 mmHg% demonstrated incremental prognostic value for all-cause mortality in all of three models, including LV global longitudinal strain (LVGLS) ≤ 20% and age, chronic kidney disease (CKD), or atrial fibrillation (AF)

**Fig. 4:**
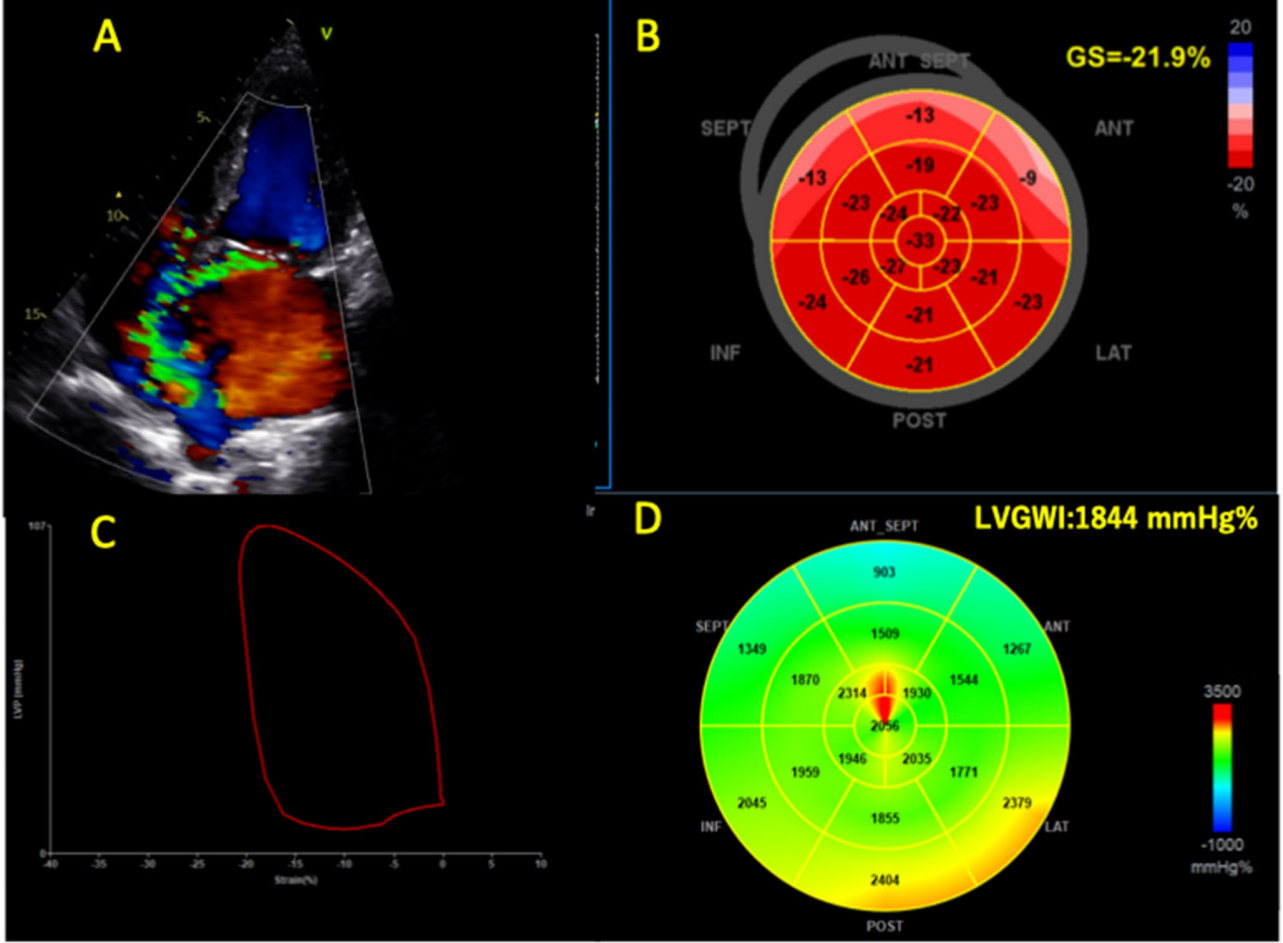
Representative case An 80-year-old patient with severe, primary mitral regurgitation due to fibroelastic deficiency. (**A**) Both left ventricular (LV) ejection fraction (76%) and LV global longitudinal strain (21.9%, **B**) were normal before surgery. LV global myocardial work index (1844 mmHg%, **C, D**), however, were already impaired

During a median follow-up of 5.0 years (interquartile range, 2.5–8.9), 27 (8.8%) patients died. Patients with LVGWI ≤ 1900 mmHg% had significantly lower survival rates compared to the group with LVGWI > 1900 mmHg% (*p* < 0.01, Fig. 2A). Those with LVGLS ≤ 20% also had significantly lower survival rates compared to the ones with LVGLS > 20% (*p* = 0.02, Fig. 2B), whereas there was no significant difference in the survival rate between patients with LVEF ≤ 60% and > 60% (*p* = 0.24, Fig. 2C). Univariate Cox regression analysis demonstrated that age, chronic kidney disease, atrial fibrillation, LVGLS ≤ 20%, LVGWI ≤ 1900 mmHg% and LVGCW ≤ 2200 mmHg% were associated with all-cause mortality. To avoid multicollinearity, we constructed two multivariate Cox regression models: model 1 included age, chronic kidney disease, and LVGWI ≤ 1900 mmHg%, whereas model 2 included age, chronic kidney disease, and LVGLS ≤ 20%. LVGWI ≤ 1900 mmHg% was independently associated with all-cause mortality, however, LVGLS ≤ 20% was not independently associated with all-cause mortality in model 2 (Table 3). LVGWI was independently associated with all-cause mortality in a multivariate model which included atrial fibrillation, systolic pulmonary artery pressure, the presence of ≥ moderate TR, TAPSE, effective regurgitant orifice area, or New York Heart Association functional class. (Supplemental Table 2) LVGWI ≤ 1900 mmHg% demonstrated incremental prognostic value for all-cause mortality in all three of the newly-constructed models, which included LVGLS ≤ 20% (*p* < 0.05) (Fig. 3) On the other hand, there were no significant differences between models 1 and 2 in terms of AUC for 5-year mortality (0.803 vs. 0.792, *p* = 0.529), C-index (0.779 vs. 0.765, *p* = 0.315) and NRI (0.184, 95%CI -1.09 to 0.501). In patients with sinus rhythm, LVGWI ≤ 1900 mmHg% was associated with all-cause mortality (*p* < 0.01). Fig. 4 shows the representative case who had normal LVGLS and impaired LVGWI. 

**Table 3:** Cox hazard model for all-cause mortality

	Univariate			Multivariate model 1			Multivariate model 2		
Characteristic	HR	95% CI	*p*-value	HR	95% CI	*p*-value	HR	95% CI	*p*-value
Age (years)	1.11	1.06, 1.17	< 0.001	1.08	1.02, 1.14	0.011	1.08	1.02, 1.14	0.011
Gender (male)	0.68	0.32, 1.45	0.320						
Tricuspid valve repair	0.37	0.15, 0.92	0.033						
Coronary artery bypass grafting	1.20	0.56, 2.56	0.639						
NYHA functional class ≥ 2	1.29	0.54, 3.06	0.563						
Arterial hypertension	0.94	0.44, 2.01	0.874						
Hypercholesterolemia	1.05	0.84, 1.30	0.678						
Chronic kidney disease	5.99	2.80, 12.8	< 0.001	2.12	0.83, 5.41	0.115	2.32	0.91, 5.94	0.078
Atrial fibrillation	2.46	1.10, 5.47	0.028						
LVEF < 60%	1.61	0.72, 3.58	0.248						
LVGLS < 20%	3.06	1.16, 8.09	0.024				2.03	0.76, 5.41	0.155
LVGWI < 1900 mmHg%	4.29	1.81, 10.1	0.001	2.94	1.23, 7.06	0.016			
LVGCW < 2200 mmHg%	3.20	1.44, 7.12	0.004						
LVGWW < 90 mmHg%	1.37	0.58, 3.23	0.478						
LVGWE < 96%	3.57	0.48, 26.4	0.211						
EROA (mm2)	1.02	1.00, 1.03	0.073						
Regurgitant volume (ml)	1.01	0.99, 1.02	0.245						
Vena contracta (mm)	1.08	0.83, 1.40	0.577						

CI, confidence interval; HR, hazard ratio; EORA, effective regurgitant orifice area; LVEF, left ventricular ejection fraction; LVGLS, left ventricular global longitudinal strain; LVGWI, left ventricular global myocardial work index; LVGCW, left ventricular global constructive work; LVGWW, left ventricular global wasted work; LVGWE, left ventricular global work efficiency; NYHA, New York Heart Association

## Discussion

The current study revealed that 42% of patients with severe, primary MR had impaired LVGWI pre-surgery. Impaired baseline LVGLS, LVGWI and LVGCW were associated with a higher risk of mortality post-surgery. While the likelihood ratio test indicated that LVGWI improves prognostic assessment, this was not corroborated by AUC, C-index or NRI.

### LV systolic function and prognosis in patients with primary MR after surgery

Pre-surgical LV dysfunction is known to lead to unfavorable outcomes and irreversible LV dysfunction post-surgery [4, 18]. In a study of 409 patients with severe primary MR undergoing MV surgery, individuals with LVEF < 60% had a lower survival rate compared to those with LVEF ≥ 60% [4]. Similarly, in a study of 335 patients with severe primary MR, impaired pre-operative LVEF predicted a high risk of post-operative LV dysfunction [19]. Based on these findings, reduced LVEF (≤ 60%) is a guideline-based indication for surgical MV intervention, even in asymptomatic patients [3]. It remains challenging, however, to assess LV systolic function in patients with MR, since valvular regurgitation creates a low-impedance pathway for LV ejection [4, 20]. This makes LVEF, which is very load-dependent, susceptible to overestimation of LV systolic function. In a recent study, which included 506 patients with severe primary MR, LVEF was not found to be associated with all-cause mortality post-surgery, which is consistent with our findings [20]. 

LVGLS is less load-dependent than LVEF, and a more sensitive maker of LV systolic dysfunction [21]. Preoperative LVGLS is a robust predictor of all-cause mortality after surgery in patients with primary MR [7, 8, 20]. Our results confirm these findings, namely that impaired LVGLS before MV surgery is associated with all-cause mortality. LVGLS, however, remains a load-dependent LV function parameter [22]. Loading conditions are dramatically altered by MV surgery due to an acute reduction of preload and an increase in afterload with closure of the low-impedance LA conduit [23, 24]. A load-independent parameter may therefore be valuable in assessing LV systolic function in patients with severe, primary MR who are being considered for surgery.

### Prognostic implications of LVMW in patients with primary MR after surgery

Echocardiography-based, non-invasive LVMW takes afterload into account when quantifying LV systolic function [9, 11]. The methodology has been validated in both preclinical and clinical models, and correlates well with clinical outcomes [11, 25]. 

The prognostic value of LVMW has been established in patients with secondary MR, where impaired LVGWI and LVGCW were associated with all-cause mortality [13]. LVGWI, measured pre-intervention, was associated with all-cause mortality in patients with severe aortic stenosis who underwent transcatheter aortic valve implantation [26]. LVGWI has also shown incremental prognostic value over LVGLS in patients with non-valvular pathologies, e.g. acute myocardial infarction and heart failure with preserved EF [27, 28]. A study of 180 patients with primary MR compared LVMW indices across different severities of MR [29]. No differences in LVGWI were found among the groups with mild, moderate, and severe primary MR. In contrast, LVGCW and LVGWW increased in relation to the severity of primary MR. No previous studies, however, have examined the relationship between LVMW indices and outcomes in patients with primary MR after surgery. Our results are the first to indicate that impaired LVGWI is independently associated with all-cause mortality in patients undergoing surgery for severe, primary MR.

The impaired LVGWI group had smaller LV end-diastolic volumes but greater LV end-systolic volumes, compared with the normal LVGWI group. Higher LV end-systolic volume reflects both anatomic and functional changes due to MR and is associated with all-cause mortality [30]. The impaired LVGWI group exhibited higher systolic pulmonary artery pressure and a higher prevalence of atrial fibrillation compared to the normal LVGWI group. While both systolic pulmonary pressure and atrial fibrillation are established prognostic markers in MR patients, LVGWI was independently associated with all-cause mortality when adjusted for systolic pulmonary artery pressure and atrial fibrillation [31]. Importantly, LVGWI has incremental prognostic value over the LVGLS.

Echocardiography-based, non-invasive LVMW may therefore be a more accurate measure of LV systolic dysfunction than LVGLS in patients presenting with severe, primary MR. Preoperative LVMW may be valuable in predicting postoperative outcomes and subsequently, determining the optimal timing for surgery in patients who suffer from severe, primary MR.

### Study limitations

The current study is limited by its retrospective, single-center, observational design. The follow-up time is limited to the medium term. We were unable to assess the all cardiac events, including heart failure hospitalization, due to the fact that patients were not routinely admitted to the Leiden University Medical Center with decompensated cardiac failure, but often to secondary medical centers. Since the number of events was limited in the current study, we were unable to adjust for all LVGWI and clinical variables which may have been associated with all-cause mortality. At present, non-invasive LVMW can be calculated by a single vendor only. The majority of patients in this study had clinical symptoms and already qualified for MV surgery on the basis of current guidelines, therefore we did not evaluate the utility of LVGWI in refining the indications for MV surgery. The measurement of blood pressure was not performed according to a standardized technique, which may have influenced LVMW calculations [32]. Since the current study is retrospective in design, we were limited as to the definition of AF in the study population. AF was therefore defined at the time of transthoracic echocardiography. We were unable to discriminate cardiac from non-cardiac death, since this distinction is not made in the municipal registries from which mortality data were obtained.

## Conclusions

In patients with severe, primary MR who underwent MV surgery, impaired pre-operative LVGWI and LVGCW, measured non-invasively, were associated with a higher risk of mortality. Although the likelihood ratio test indicates incremental prognostic value of LVGWI over the model including LVGLS, neither the C-index nor NRI demonstrated a statistically significant incremental value. This highlights the need for further studies to validate the prognostic utility of LVGWI.

## Electronic supplementary material

Below is the link to the electronic supplementary material.

Supplementary Material 1

## Data Availability

No datasets were generated or analysed during the current study.
