# Supplementary material for: Prognostic impact of left ventricular myocardial work in patients undergoing surgery for primary mitral regurgitation
Source: Int J Cardiovasc Imaging. 2025 Mar 29;41(5):991–1000. doi: 10.1007/s10554-025-03386-x (PMC12075355; doi:10.1007/s10554-025-03386-x)
Supplement: Supplementary file 1 — Supplementary Material 1 [file 10554_2025_3386_MOESM1_ESM.docx]

**Supplemental table 1. Baseline clinical and echocardiographic characteristics, stratified according to outcome**

| **Characteristic** | **Overall**  **population,**  **N = 306** | **Event (-),**  **N = 279** | **Event (+),**  **N = 27** | **p-value** |
| --- | --- | --- | --- | --- |
| Age (years) | 63 (12) | 62 (11) | 73 (7) | <0.001 |
| Gender (male) | 209 (68%) | 194 (70%) | 15 (56%) | 0.136 |
| Etiology of mitral regurgitation |  |  |  | 0.008 |
| Fibroelastic deficiency | 164 (54%) | 143 (51%) | 21 (78%) |  |
| Advanced myxomatous disease | 142 (46%) | 136 (49%) | 6 (22%) |  |
| NYHA functional class ≥II | 229 (75%) | 209 (75%) | 20 (74%) | 0.924 |
| Arterial hypertension | 121 (40%) | 109 (39%) | 12 (44%) | 0.585 |
| Hypercholesterolemia | 19 (12%) | 15 (9.8%) | 4 (44%) | 0.009 |
| Diabetes mellitus | 9 (2.9%) | 9 (3.2%) | 0 (0%) | >0.999 |
| Chronic kidney disease | 55 (18%) | 40 (14%) | 15 (56%) | <0.001 |
| Atrial fibrillation | 48 (16%) | 39 (14%) | 9 (33%) | 0.022 |
| Systolic blood pressure (mmHg) | 135 (19) | 136 (19) | 132 (19) | 0.316 |
| Diastolic blood pressure (mmHg) | 78 (11) | 78 (12) | 77 (11) | 0.833 |
| Heart rate (/min) | 75 (16) | 74 (16) | 80 (20) | 0.161 |
| Cardiac surgery |  |  |  |  |
| Tricuspid valve repair | 148 (48%) | 133 (48%) | 15 (56%) | 0.434 |
| Coronary artery bypass grafting | 41 (13%) | 38 (14%) | 3 (11%) | >0.999 |
| Echocardiographic parameters |  |  |  |  |
| LVEDV index (ml/m2) | 75 (20) | 76 (20) | 67 (16) | 0.012 |
| LVESV index (ml/m2) | 27 (10) | 27 (10) | 25 (10) | 0.344 |
| LVEF (%) | 64 (8) | 64 (8) | 63 (10) | 0.570 |
| LVGLS (%) | 19 (4) | 19 (4) | 17 (4) | 0.004 |
| LAVI (ml/m2) | 56 (23) | 56 (23) | 57 (23) | 0.946 |
| LVGWI (mmHg%) | 1979 (539) | 2010 (539) | 1661 (429) | <0.001 |
| LVGCW (mmHg%) | 2360 (585) | 2392 (582) | 2030 (512) | 0.002 |
| LVGWW (mmHg%) | 151 (104) | 150 (105) | 161 (101) | 0.600 |
| LVGWE (%) | 92.3 (4.4) | 92.4 (4.2) | 90.6 (5.3) | 0.094 |
| EROA (mm2) | 49 (20) | 48 (20) | 55 (27) | 0.243 |
| Regurgitant volume (ml) | 62 (24) | 62 (24) | 67 (22) | 0.230 |
| Vena contracta (mm) | 7.1 (1.6) | 7.0 (1.6) | 7.6 (1.5) | 0.067 |
| TAPSE (mm) | 23 (5) | 24 (4) | 21 (5) | 0.004 |
| Systolic PAP (mmHg) | 38 (16) | 37 (15) | 46 (19) | 0.019 |
| TR ≥ moderate | 71 (23%) | 57 (20%) | 14 (52%) | <0.001 |

Values are expressed as mean (±standard deviation). EORA, effective regurgitant orifice area; LAVI, left atrial volume index; LVEDV, left ventricular end-diastolic volume; LVEF, left ventricular ejection fraction; LVESV, left ventricular end-systolic volume; LVGLS, left ventricular global longitudinal strain; left ventricular global longitudinal strain; LVGWI, left ventricular global myocardial work index; LVGCW, left ventricular global constructive work; LVGWW, left ventricular global wasted work; LVGWE, left ventricular global work efficiency; NYHA, New York Heart Association; PAP, pulmonary artery pressure; TAPSE, tricuspid annular plane systolic excursion; TR, tricuspid regurgitation

**Supplemental table 2. Multivariate Cox regression models for all-cause mortality, adjusted for atrial fibrillation, systolic pulmonary artery pressure, moderate TR, TAPSE, EROA, or NYHA functional class**

|  | **Multivariate model** | | |
| --- | --- | --- | --- |
| **Characteristic** | **HR** | **95% CI** | **p-value** |
| Model 1 |  |  |  |
| LVGWI <1900 mmHg% | 3.83 | 1.54, 9.52 | 0.004 |
| Atrial fibrillation | 1.43 | 0.61, 3.33 | 0.407 |
| Model 2 |  |  |  |
| LVGWI <1900 mmHg% | 3.51 | 1.47, 8.36 | 0.005 |
| Systolic PAP (mmHg) | 1.03 | 1.00, 1.05 | 0.023 |
| Model 3 |  |  |  |
| LVGWI <1900 mmHg% | 3.48 | 1.43, 8.44 | 0.006 |
| TR ≥ moderate | 2.33 | 1.07, 5.07 | 0.033 |
| Model 4 |  |  |  |
| LVGWI <1900 mmHg% | 3.36 | 1.36, 8.31 | 0.009 |
| TAPSE (mm) | 0.92 | 0.84, 1.00 | 0.047 |
| Model 5 |  |  |  |
| LVGWI <1900 mmHg% | 3.57 | 1.47, 8.66 | 0.005 |
| EROA (mm^2^) | 1.01 | 0.99, 1.03 | 0.228 |
| Model 6 |  |  |  |
| LVGWI <1900 mmHg% | 4.30 | 1.80, 10.30 | 0.001 |
| NYHA functional class < 2 | 0.98 | 0.41, 2.36 | 0.970 |

CI, confidence interval; EORA, effective regurgitant orifice area; LVGWI, left ventricular global myocardial work index; NYHA, New York Heart Association; PAP, pulmonary artery pressure; TAPSE, tricuspid annular plane systolic excursion; TR, tricuspid regurgitation
